# Comparison of Left Ventricular Global Longitudinal Strain and Left Ventricular Ejection Fraction in Acute Respiratory Failure Patients Requiring Invasive Mechanical Ventilation

**DOI:** 10.3390/jcdd11110339

**Published:** 2024-10-24

**Authors:** Zubair Bashir, Feven Ataklte, Shuyuan Wang, Edward W. Chen, Vishnu Kadiyala, Charles F. Sherrod, Phinnara Has, Christopher Song, Corey E. Ventetuolo, James Simmons, Philip Haines

**Affiliations:** 1 Department of Cardiology, University of Texas Medical Branch, Galveston, TX 77555, USA; 2 Department of Cardiology, Alpert Medical School of Brown University, Providence, RI 02903, USA; 3 Department of Cardiology, First Affiliated Hospital of Nanjing Medical University, Nanjing 210029, China; 4 Department of Ultrasound Medicine, Union Hospital, Tongji Medical College, Huazhong University of Science and Technology, Wuhan 430022, China; 5 Department of Internal Medicine, Yale School of Medicine, New Haven, CT 06510, USA; 6 Department of Cardiology, Healthcare Institute for Innovations in Quality, University of Missouri-Kansas City, Kansas City, MO 64110, USA; 7 Saint Luke’s Mid America Heart Institute, Kansas City, MO 64111, USA; 8 Lifespan Biostatistics, Epidemiology and Research Design, Rhode Island Hospital, Providence, RI 02903, USA; 9 Division of Pulmonary, Critical Care, and Sleep Medicine, Alpert Medical School of Brown University, Providence, RI 02903, USA; 10 Department of Health Services, Policy & Practice, Brown University School of Public Health, Providence, RI 02903, USA

**Keywords:** left ventricular global longitudinal strain, left ventricular ejection fraction, acute respiratory failure, invasive mechanical ventilation

## Abstract

Left ventricular (LV) dysfunction is associated with poor clinical outcomes in acute respiratory failure (ARF). This study evaluates the efficacy of LV strain in detecting LV dysfunction in ARF patients requiring invasive mechanical ventilation (IMV) compared to conventionally measured left ventricular ejection fraction (LVEF). ARF patients requiring IMV who had echocardiography performed during MICU admission were included. LV global longitudinal strain (LVGLS) and LVEF were measured retrospectively using speckle tracking (STE) and traditional transthoracic echocardiography (TTE), respectively, by investigators blinded to the status of IMV and clinical data. The cohort was divided into three groups: TTE during IMV (TTE-IMV), before IMV (TTE-bIMV), and after IMV (TTE-aIMV). Multivariable regression models, adjusted for illness severity score, chronic cardiac disease, acute respiratory failure etiology, body mass index, chronic obstructive pulmonary disease, and obstructive sleep apnea, evaluated associations between LV function parameters and the presence of IMV. Among 376 patients, TTE-IMV, TTE-bIMV, and TTE-aIMV groups constituted 223, 68, and 85 patients, respectively. The median age was 65 years (IQR: 56–74), with 53.2% male participants. Adjusted models showed significantly higher LVGLS in groups not on IMV at the time of TTE (TTE-bIMV: β = 4.19, 95% CI 2.31 to 6.08, *p* < 0.001; TTE-aIMV: β = 3.79, 95% CI 2.03 to 5.55, *p* < 0.001), while no significant differences in LVEF were observed across groups. In a subgroup analysis of patients with LVEF ≥55%, the significant difference in LVGLS among the groups remained (TTE-bIMV: β = 4.18, 95% CI 2.22 to 6.15, *p* < 0.001; TTE-aIMV: β = 3.45, 95% CI 1.50 to 5.40, *p* < 0.001), but was no longer present in those with LVEF < 55%. This suggests an association between IMV and lower LVGLS in ARF patients requiring IMV, indicating that LVGLS may be a more sensitive marker for detecting subclinical LV dysfunction compared to LVEF in this population. Future studies should track and assess serial echocardiography data in the same cohort of patients pre-, during, and post-IMV in order to validate these findings and prognosticate STE-detected LV dysfunction in ARF patients requiring IMV.

## 1. Introduction

Left ventricular (LV) strain evaluates LV function by quantifying myocardial deformation in response to internal and external stimuli. Speckle tracking echocardiography (STE) is the most commonly used technique to measure strain, with global longitudinal strain (GLS) being the primary strain parameter reported in clinical practice due to its strong association with clinical outcomes [1,2,3]. GLS measures changes in the length of myocardial fibers along the longitudinal axis from diastole to systole [4]. The normal range for global longitudinal strain is 18.0% to 21.5%, with inter-vendor variations up to 3.7% strain units [5]. Additional strain parameters include circumferential strain and radial strain, which measure changes in myocardial length and ventricular wall thickness along the circumferential and radial axes, respectively [4]. These parameters are not currently used in clinical practice.

GLS is the most robust and reproducible measure among the various myocardial strain measures [5]. It assesses the longitudinally oriented subendocardial myocardial fibers, which are highly vulnerable to myocardial disease. As a result, GLS can effectively detect myocardial dysfunction not appreciated by current standard echocardiography, such as left ventricular ejection fraction (LVEF). This myocardial systolic contractile dysfunction detected by assessing GLS is highly clinically relevant in diverse patient populations including asymptomatic patients and patients with symptomatic cardiac disease [2,3].

Invasive mechanical ventilation (IMV), an important therapeutic modality in critically ill patients, is known to exert hemodynamic effects on the heart [6]. We have previously published on the effects of IMV on right ventricular (RV) function using strain assessment [7,8]. We showed that RV-GLS and FWLS is a feasible measure to assess RV function in patients requiring IMV and is more sensitive in detecting changes in RV function compared to TAPSE [7,8]. However, data on the utilization of left ventricular GLS (LVGLS) to assess LV function among critical care patients requiring IMV are limited. Understanding the changes in LV function during acute respiratory failure while on and off IMV may inform decisions that contribute to more comprehensive patient care, especially in patients with normal baseline cardiac function. In this current study, we aimed to assess LV function in critically ill patients with a primary diagnosis of acute respiratory failure (ARF) who require IMV using STE-measured GLS. We hypothesize that LVGLS is a feasible measure for assessing LV function in ARF patients requiring IMV, and that it can identify subtle changes in LV function that remain undetected by traditional LVEF assessments.

## 2. Materials and Methods

This retrospective observational study received approval from the Lifespan institutional review boards of both Rhode Island Hospital and The Miriam Hospital (approval reference 201018 45CFR). Informed consent was waived due to the retrospective nature of the study [7,8].

All adult patients diagnosed with ARF necessitating IMV for over 24 h and who underwent transthoracic echocardiography (TTE) during their admission to a medical intensive care unit (MICU) between January 2010 and January 2018 were included. Three distinct groups were formed based on the timing of TTE in relation to IMV initiation: TTE during IMV (TTE-IMV), TTE before IMV (TTE-bIMV), and TTE after IMV (TTE-aIMV). All participants underwent at least one TTE during their MICU stay and were sorted based on the first interpretable echocardiogram if none were performed during IMV [9]. For patients who had TTEs while not on IMV (TTE-bIMV and TTE-aIMV), the echocardiogram closest to the initiation of IMV was utilized for analysis. All the participants were included in only one of the above-mentioned groups. Exclusion criteria included pregnancy, intubation for reasons other than ARF, LVEF below 40%, more than moderate aortic or mitral valve disease, presence of prosthetic heart valves, pericardial effusion with tamponade physiology, congenital heart pathology, or inadequate image quality for STE analysis, including inappropriate tracking of more than one wall of the left ventricle. Some data from the TTE-IMV group had been previously reported by this research group in earlier publications [7,8]. The study adheres to the Strengthening the Reporting of Observational Studies in Epidemiology (STROBE) framework for observational studies [9].

### 2.1. Clinical Variables

Clinical and outcome data were extracted from the electronic medical records for each participant, encompassing demographics, social and medical histories, ARF etiology, illness severity score variables, vasopressor and inotrope usage, and echocardiography parameters. To compute the illness severity score (Acute Physiology and Chronic Health Evaluation [APACHE] II), all relevant clinical data were gathered closest to the timing of TTE, within a 10 h window. In instances where variables necessary for assessing illness severity were unavailable, the lowest attainable value was utilized. These data were documented in the Research Electronic Data Capture (RedCap) database.

### 2.2. Echocardiography Measurements

Standard 2-D measurements of LVEF were retrieved from the clinical echocardiography reports interpreted by board-certified cardiologists. However, STE measurements were independently obtained by two trained investigators (S.W. and Philip Haines), who were blinded to the original echocardiography reports, clinical data, and status of IMV.

All images were analyzed using two-dimensional speckle tracking software (TomTec Imaging Systems, Chicago, IL, USA) in accordance with guidelines from the European Association of Cardiovascular Imaging (EACVI) and the American Society of Echocardiography (ASE) [10]. Endocardial borders were delineated in the end-systolic frame from cine loops in the apical 4-, 3-, and 2-chamber views of the LV, with speckles automatically tracked frame-by-frame throughout the cardiac cycle. Manual adjustments were made to the boundaries if tracking was inadequate. LVEF, end-diastolic volume (EDV), end-systolic volume (ESV), and endocardial global longitudinal strain (Endo-GLS) were calculated. Endo-GLS was determined by averaging peak systolic strain across all 16 segments. Speckle tracking quality was assessed visually and reported as a tracking feasibility score (TFS), with a score of 3 indicating all segments tracking appropriately, score of 2 indicating all but one segment tracking appropriately, and score of 1 indicating more than one segment not tracking appropriately. Images with a TFS less than 2 were excluded from statistical analysis. We reported absolute values for the LVGLS to simplify interpretation as these are originally reported in negative values.

### 2.3. Statistical Analysis

Continuous variables were presented as mean and standard deviation for normally distributed data or as median and interquartile range (IQR) for non-normally distributed data. Group comparisons were conducted using the Kruskal–Wallis test with Bonferroni correction for multiple comparisons. Categorical data were expressed as frequency and percentages and compared using Pearson’s chi-squared or Fisher’s exact test where appropriate. Linear regression models were used to compare measures of LV function among the TTE-IMV, TTE-bIMV, and TTE-aIMV groups. Multivariable models assessed the association between IMV and LV function measures across the three groups, adjusting for covariates that were significantly different between the reference (TTE-IMV) and the other two groups (TTE-bIMV and TTE-aIMV), which includes chronic cardiac disease (defined as those with ischemic and non-ischemic cardiomyopathy, valvular heart disease, chronic arrhythmias, and chronic pericarditis), illness severity (APACHE II score), documented etiology of acute respiratory failure, body mass index (BMI), chronic obstructive pulmonary disease (COPD), and obstructive sleep apnea (OSA). The TTE-IMV group served as reference, and differences in means between the reference and the other two groups were reported as β-coefficients. A two-sided *p*-value of less than 0.05 was considered statistically significant. All statistical analyses were conducted using Stata/MP 16.1 (College Station, TX, USA).

## 3. Results

**Figure 1:**
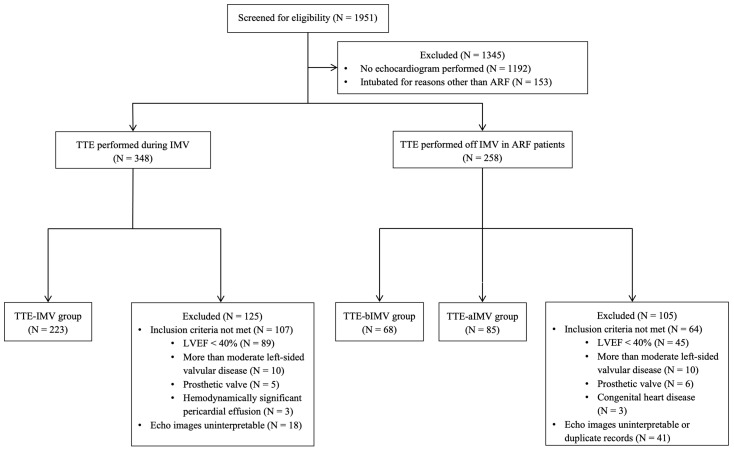
Flowchart of the analytic sample.

Of the 376 patients admitted to the MICU with ARF necessitating IMV and meeting the inclusion criteria, 223, 68, and 85 participants were allocated to the TTE-IMV, TTE-bIMV, and TTE-aIMV groups, respectively (Figure 1). The median age of the entire cohort was 65 years (interquartile range: 56–74), with males comprising 53.2%. While the clinical characteristics of the reference and other two groups were generally comparable, notable differences were observed in median body mass index, incidence of shock requiring vasopressor therapy, number of pressor used, APACHE II score, chronic lung disease, and etiology of ARF (Table 1). Pneumonia emerged as the predominant cause of ARF across all groups with a significantly higher prevalence in the TTE-IMV group. A significantly higher prevalence of cardiac arrest was also found in the TTE-IMV group as compared to the other two groups, whereas a higher prevalence of acute COPD exacerbation as an etiology of ARF was found in the TTE-aIMV group as compared to the reference group (TTE-IMV). Furthermore, a history of COPD and OSA was more prevalent in the TTE-aIMV group as compared to the TTE-IMV group. As anticipated, the TTE-IMV group exhibited a higher median total APACHE II score at the time of TTE compared to the other groups (Table 1). In terms of timing, the TTE-IMV group underwent echocardiography approximately 1.4 ± 1.9 days after intubation. Conversely, individuals in the TTE-bIMV and TTE-aIMV groups received echocardiograms an average of 3.6 ± 3.9 days before initiation of IMV and approximately 3.2 ± 2.7 days after extubation, respectively. The median peak end-expiratory pressure (PEEP) among the participants in the TTE-IMV group at the time of TTE was 8 (IQR: 5–10) cmH2O. A subgroup analysis of the TTE-IMV group showed a significant positive association between PEEP and LVGLS (r = 0.19, *p* = 0.004), as opposed to LVEF.

### 3.1. Association Between Invasive Mechanical Ventilation and Measures of LV Function

The TTE-IMV group had significantly worse LV function compared to the TTE-bIMV and TTE-aIMV groups as assessed by LVGLS (median [Q1–Q3]: 15.5 [11.7–18.7] vs. 19.4 [15.1–23.9] vs. 18.7 [14.7–22.7], *p* < 0.001 for both comparisons). No significant difference in the LV function was found by LVEF among all the three groups (median [Q1–Q3]: 60 [55–65] vs. 64 [55–70] vs. 65 [55–65], *p* = 0.09 and *p* = 0.29, respectively) (Table 2).

In unadjusted multivariable regression analysis assessing the association between IMV and LV function, we found that the differences in means of LVGLS between the reference group (TTE-IMV) and the other two groups showed significantly higher LVGLS in the two groups not on IMV at the time of echocardiography (TTE-bIMV: β = 3.74, 95% CI 2.17 to 5.30, *p* < 0.001; TTE-aIMV: β = 3.18, 95% CI 1.80 to 4.56, *p* < 0.001). Conversely, LVEF failed to identify any significant differences in the LV function between the reference and the other two groups (TTE-bIMV: β = 2.75, 95% CI 0.002 to 5.49, *p* = 0.050; TTE-aIMV: β = 1.68, 95% CI −0.73 to 4.09, *p* = 0.17).

After adjusting for chronic cardiac disease, APACHE II score, etiology of acute respiratory failure, BMI, COPD, and OSA, the differences in means of LVGLS between the reference group and the other two groups continued to show significantly higher LVGLS in the groups not on IMV at the time of echocardiography (TTE-bIMV: β = 4.19, 95% CI 2.31 to 6.08, *p* < 0.001; TTE-aIMV: β = 3.79, 95% CI 2.03 to 5.55, *p* < 0.001). Again, no significant differences were found in LVEF between the reference and the other two groups (TTE-bIMV: β = 2.12, 95% CI −1.16 to 5.42, *p* = 0.21; TTE-aIMV: β = 2.12, 95% CI −1.04 to 4.48, *p* = 0.19) (Table 3).

### 3.2. Subgroup Analysis

Among the participants with LVEF ≥ 55% as reported in the clinical echocardiography reports, a significantly lower LVGLS was found in the TTE-IMV group as compared to the TTE-bIMV and TTE-aIMV groups, respectively (median [Q1–Q3]: 16.6 [13.2–19.6] vs. 19.8 [17.1–24.2] vs. 19.6 [16.2–23.4], *p* < 0.001 for both comparisons). There was no significant difference in LVEF between the TTE-IMV group (median [Q1–Q3]: 65 [60–70] vs. 65 [60–70] vs. 65 [60–65], *p* = 0.67 and *p* = 1.00, respectively) and the other two groups (Table 4).

In unadjusted multivariable regression analysis assessing the association between IMV and LV function among participants with LVEF ≥ 55%, we found that the differences in the means of LVGLS indicated a significantly higher LVGLS in TTE-bIMV (β = 3.34, 95% CI 1.68 to 4.99, *p* < 0.001) and TTE-aIMV (β = 3.05, 95% CI 1.59 to 4.51, *p* < 0.001) groups as compared to the reference group.

After adjusting for chronic cardiac disease, APACHE II score, etiology of acute respiratory failure, BMI, COPD, and OSA, the differences in means continued to show significantly higher LVGLS in the groups not on IMV at the time of echocardiography as compared to the reference group (TTE-bIMV: β = 4.18, 95% CI 2.22 to 6.15, *p* < 0.001; TTE-aIMV: β = 3.45, 95% CI 1.50 to 5.40, *p* < 0.001) (Table 5).

Interestingly, this trend was not seen among the participants with LVEF < 55%. There were no significant differences in LVGLS (median [Q1–Q3]: 12.2 [9.2–14.8] vs. 12.6 [10.1–16.7] vs. 14.1 [9.2–15.8], *p* = 0.23 and *p* = 0.19, respectively) or LVEF (median [Q1–Q3]: 45 [40–50] vs. 45 [40–50] vs. 45 [42.5–45], *p* = 0.47 and *p* = 0.37, respectively) among the three groups, but notably all groups had abnormally low LVGLS by most current definitions (Supplementary Table S1).

## 4. Discussion

In the present study involving a population of 376 heterogeneous patients with ARF, we found that LV strain assessment is feasible and practical among the critically ill mechanically ventilated patients in the intensive care setting. We observed significant differences in LV function associated with the timing of IMV, most notably that patients undergoing echocardiography while on IMV were associated with lower LVGLS compared to those who had their echocardiogram performed before or after IMV, even after adjusting for potential confounders. In comparison, there were no significant differences in LVEF between these respective groups, highlighting that LVGLS may be a more sensitive marker for detecting subclinical cardiac dysfunction than traditionally measured LVEF.

To our knowledge, only one small prior study has specifically assessed LV strain among patients with respiratory failure requiring IMV. In this study, Franchi et al. [11] assessed 20 patients admitted to a mixed ICU requiring IMV for hypoxia to determine the effects of positive end-expiratory pressure (PEEP) on four-chamber longitudinal strain [11]. Our study findings were different from Franchi et al. [3], who reported that LVGLS and LVEF remained stable with PEEP titration as opposed to right atrial (RA), RV, and left atrial (LA) strains which showed a significant decrease. They proposed that the changes in cardiac function seen during IMV and PEEP are related to loading changes instead of actual LV contractility changes [11]. However, given the small sample size, their power to detect small (but potentially clinically significant) differences in LVGLS may have been limited.

Other studies that assessed the effects of IMV on LV function were in the setting of general anesthesia (GA) for surgical procedures, reporting similar findings to ours [12,13]. Dalla et al. [12] looked at the effects of GA and positive pressure ventilation (PPV) on right and left ventricular loading conditions among 23 patients with no underlying myocardial dysfunction. A decrease in LVGLS but not LVEF was observed in these patients after anesthesia induction and application of PPV [12]. Moreover, Cinotti et al. [13] reported the effects of IMV and general anesthesia on LVGLS among American Society of Anesthesia (ASA) class I–II patients with a baseline normal LV function undergoing routine surgical procedures. They found that LVGLS was reduced after GA and IMV in patients without cardiovascular co-morbidities but remained within the normal range. They also found the LVGLS returned to the baseline values after extubation [13]. Neither of these studies was able to elucidate whether the reduction in GLS was due to GA or PPV alone. Our study, which is much larger than the two aforementioned studies, extends the utility of assessing LV function with LVGLS beyond patients undergoing GA; therefore, our findings promote the more general clinical utility of LVGLS in patients with ARF requiring IMV.

The observed findings in our study may have a biological basis, given the impact of IMV on cardiac and respiratory physiology. The hemodynamic effects of IMV include changes in intrathoracic pressures and pulmonary vascular resistance, which in turn can impact the cardiac pre-load and afterload and possibly cause compression of the heart in the cardiac fossa similar to the effects seen in cardiac tamponade [6]. IMV increases the intrathoracic pressure, which reduces the pre-load. In addition, changes in lung volumes during inspiration with mechanical ventilation may cause a decrease in pulmonary vascular resistance by recruiting more alveoli as well as a decrease in LV transmural pressure, thus reducing LV afterload; this may offset some of the pre-load reducing effects of PPV [6]. This decrease in afterload may explain the improvement in LV function, as detected by LVGLS, with increasing PEEP. However, there may be a biphasic pattern of LV function changes with increasing PEEP, as the median PEEP in our cohort was mild to moderate. Therefore, the initial improvements in LV function following initiation of IMV may be followed by worsening in LV function as PEEP increases beyond unknown higher threshold. Furthermore, these changes in LV function may be influenced by multiple MV parameters, such as the mode of mechanical ventilation, tidal volume, respiratory rate, airflow obstruction, peak inspiratory pressure, plateau pressure, and inspiratory flow rate, which may ultimately determine the effects on LV function. Nonetheless, LVGLS was able to capture these subtle changes in LV function in a large heterogeneous population on IMV, unlike LVEF.

Although our study suggests that LVGLS is able to detect subclinical changes in LV function that may be partly influenced by the presence of IMV during TTE as compared to LVEF, the TTE-IMV had a significantly high number of patients with cardiac arrest, which may have influenced the LV function independent of IMV status. However, the results remained significant after adjusting for this known confounder. Moreover, LVEF failed to detect any absolute LV dysfunction in this population and also any significant changes in LV function between the TTE-IMV group and the other two. However, LVGLS detected LV dysfunction in the TTE-IMV group which could partly be due to IMV or underlying cardiac pathology or both. It also identified significant changes in LV function between the reference and the other two groups in this subgroup. Nonetheless, it is uncertain whether the LV dysfunction is long-lasting or a temporary effect of IMV influenced by underlying myocardial dysfunction as modulated by the aforementioned hemodynamic changes. Perhaps the normal LVEF and LVGLS in the groups that are not on IMV suggest that these changes are reversible, but the lack of repeated TTEs in this retrospective study did not allow us to evaluate this. Future studies assessing data on pre- and post-IMV LVGLS in individual subjects are needed to confirm this interpretation.

In addition, in the subgroup analysis of patients with LVEF > 55%, the discriminatory ability of LVGLS to detect LV dysfunction persisted, but it was diminished in those with LVEF < 55%, with LVGLS notably decreased in all groups. This is likely due to the curvilinear relationship between GLS and LVEF in patients with preserved EF, as opposed to the more linear relationship seen in those with reduced EF [14]. These findings underscore the utility of LVGLS in detecting subtle systolic dysfunction, even in patients traditionally considered to have “normal systolic function” with LVEF ≥ 55%. Furthermore, LVGLS remains a more powerful predictor of outcomes than LVEF in the range of 40–55%, demonstrating its added prognostic value across varying degrees of LV function [1,2].

The strengths and limitations of the study merit some consideration. Regarding limitations, first, this is a single-center retrospective study, which may limit generalizability. Second, the retrospective design hampers our ability to evaluate the temporal sequence between LV dysfunction and factors such as illness severity and IMV. Despite adjusting for potential confounders, the observational nature of the study means that unmeasured residual confounding variables may influence the estimates. Third, there was no uniform single time point for measuring illness severity scores in all three groups, and these scores were not repeatedly assessed during the patients’ hospital stays to determine the highest severity of illness. Additionally, not all clinical data for illness severity by APACHE II score were available for review, leading to potential underestimation of illness severity across all groups. However, by measuring illness severity score at the time of TTE, we felt this reflected the active clinical disease potentially affecting cardiac function at the time of strain measurements. In an ideal environment, multiple echocardiographic measurements on the same patients with serial assessment of APACHE II scores before, during, and after the IMV would have helped in consolidating the interpretation of the study. Lastly, we did not assess the interobserver correlation coefficient in this study. However, the strain analysis was measured by the same investigators who previously performed strain measurements in our study on RV strain, which demonstrated moderate to good interobserver reliability between the investigators [8].

Regarding strengths, first, this study is much larger than prior studies performed on similar topics. Second, while various software options for STE are available, we used TomTec for the entire cohort, which is a leading vendor-independent software package for performing STE. Third, LVGLS measurements were independently obtained and confirmed by two trained investigators. Fourth, the investigators were blinded to the original TTE reports, clinical data, and the status of IMV. Furthermore, we adjusted for known potential confounders including APACHE score, which is a well-accepted measure of illness severity in critically ill patients. We also performed a few sensitivity analyses that confirmed our main findings. Overall, our study adds to the current dearth of literature on this topic, serving as a source of hypothesis generation and informing the design of future prospective longitudinal studies which we feel would be feasible in an ICU setting.

## 5. Conclusions

Our study showed evidence of LV systolic dysfunction as measured by LVGLS but not LVEF among ARF patients requiring IMV. We found that LVGLS was able to detect LV dysfunction among participants with normal LVEF, suggesting that it may be more sensitive in detecting subtle yet significant changes in LV function during ARF with IMV that are undetectable using conventional parameters. Future investigations should focus on elucidating the primary driver of LV dysfunction in this population while evaluating the clinical significance of GLS-detected LV dysfunction.

## Figures and Tables

**Table 1:** Baseline characteristics of total cohort who had transthoracic echocardiogram (TTE) performed while on invasive mechanical ventilation (TTE-IMV), before invasive mechanical ventilation (TTE-bIMV), and after being on invasive mechanical ventilation (TTE-aIMV).

N = 376	Total Cohort (N = 376)	TTE-IMV (N = 223)	*p*-Value (IMV vs. bIMV)	TTE-bIMV (N = 68)	*p*-Value (IMV vs. aIMV)	TTE-aIMV (N = 85)
Age	(n = 375)	(n = 223)		(n = 67)		(n = 85)
Median (Q1–Q3)	65 (56–74)	65 (55–74)	0.16	71 (57–76)	1.00	63 (57–74)
Sex						
Male	200 (53.2)	121 (54.3)	0.65	39 (57.4)	0.26	40 (47.1)
Documented smoking history	(n = 354)	(n = 201)		(n = 68)		(n = 85)
Yes	230 (64.9)	126 (62.7)	0.07	51 (75.0)	0.96	53 (62.4)
BMI (kg/m^2^)						
Median (Q1–Q3)	28.9 (24.3–35.6)	29.7 (25.2–37.1)	0.17	27.8 (22.9–35.3)	0.03	28.2 (22.8–33.1)
Chronic lung disease *						
Chronic obstructive lung disease	138 (36.7)	71 (31.8)	0.33	26 (38.2)	0.01	41 (48.2)
Asthma	28 (7.5)	13 (5.8)	0.39	6 (8.8)	0.15	9 (10.6)
Interstitial lung disease	10 (2.7)	5 (2.2)	0.69	1 (1.5)	0.26	4 (4.7)
Cystic fibrosis	1 (0.27)	1 (0.45)	--	0 (--)	--	0 (--)
Pulmonary hypertension	18 (4.8)	12 (5.4)	0.42	2 (2.9)	0.81	4 (4.7)
Obstructive sleep apnea	51 (13.6)	22 (9.9)	0.08	12 (17.7)	0.02	17 (20.0)
History of primary lung cancer	(n = 375)	(n = 222)		(n = 68)		(n = 85)
Yes	19 (5.1)	9 (4.1)	0.53	4 (5.9)	0.28	6 (7.1)
Chronic cardiac disease *^,^^	280 (74.5)	159 (71.3)	0.02	58 (85.3)	0.62	63 (74.1)
Chronic renal disease *^,#^	47 (12.5)	32 (14.4)	0.39	7 (10.3)	0.25	8 (9.4)
Shock requiring pressor support	249 (66.2)	162 (72.7)	0.21	44 (64.7)	<0.001	43 (50.6)
Number of pressors used	(n = 249)	(n = 162)	0.93	(n = 44)	0.004	(n = 43)
1	125 (50.2)	73 (45.1)		21 (47.7)		31 (72.1)
2	84 (33.7)	58 (35.8)		16 (36.4)		10 (23.3)
3	40 (16.1)	31 (19.1)		7 (15.9)		2 (4.7)
Inotropes	(n = 375)	(n = 223)		(n = 68)		(n = 84)
Yes	18 (4.8)	11 (4.9)	0.45	5 (7.4)	0.33	2 (2.4)
Total APACHE II score	(n = 364)	(n = 223)		(n = 64)		(n = 77)
Median (Q1–Q3)	18 (12–24)	22 (17–27)	<0.001	13.5 (10–19)	<0.001	11 (6–15)
Etiology of acute respiratory failure	(n = 375)	(n = 222)	0.001	(n = 68)	<0.001	(n = 85)
Pneumonia	120 (32.0)	82 (36.9)		19 (27.9)		19 (22.4)
Pulmonary edema	64 (17.1)	34 (15.3)		17 (25.0)		13 (15.3)
Cardiac arrest	60 (16.0)	46 (20.7)		2 (2.9)		12 (14.1)
Aspiration	54 (14.4)	29 (13.1)		15 (22.1)		10 (11.8)
Acute exacerbation COPD	24 (6.4)	6 (2.7)		3 (4.4)		15 (17.7)
Encephalopathy	15 (4.0)	5 (2.3)		4 (5.9)		6 (7.1)
Other	38 (10.1)	20 (9.0)		8 (11.8)		10 (11.8)
ARDS diagnosed	(n = 369)	(n = 223)		(n = 62)		(n = 84)
Yes	37 (10.0)	21 (9.4)	0.14	10 (16.1)	0.53	6 (7.1)

Categorical data are N (%). * does not sum to 100%. ^ includes ischemic and non-ischemic cardiomyopathy, valvular heart disease, chronic arrhythmias, or chronic pericarditis. ^#^ includes > stage III or chronic hemodialysis.

**Table 2:** Echocardiography parameters comparing ARF patients with TTE performed at different time points relative to presence of IMV.

N = 376	Total Cohort (N = 376)	TTE-IMV (N = 223)	TTE-bIMV (N = 68)	*p*-Value ^1^ (IMV vs. bIMV)	TTE-aIMV (N = 85)	*p*-Value ^1^ (IMV vs. aIMV)
LVGLS *	(n = 365)	(n = 221)	(n = 63)		(n = 81)	
Median (Q1–Q3)	16.8 (12.9–20.8)	15.5 (11.7–18.7)	19.4 (15.1–23.9)	<0.001	18.7 (14.7–22.7)	<0.001
LVEF	(n = 364)	(n = 213)	(n = 66)		(n = 85)	
Median (Q1–Q3)	60 (55–65)	60 (55–65)	64 (55–70)	0.09	65 (55–65)	0.29

^1^ Kruskal–Wallis test with Bonferroni correction for multiple comparisons. * Absolute strain values are reported with lower values indicating worse strain.

**Table 3:** Unadjusted and adjusted analysis for the echocardiography parameters comparing ARF patients with TTE performed at different time points relative to presence of IMV.

	Unadjusted β (95% CI)	*p*-Value	Adjusted β ^†^ (95% CI)	*p*-Value
LVGLS				
TTE during	Reference	Reference	Reference	Reference
TTE before IMV	3.74 (2.17, 5.30)	<0.001	4.19 (2.31, 6.08)	<0.001
TTE after IMV	3.18 (1.80, 4.56)	<0.001	3.79 (2.03, 5.55)	<0.001
LVEF				
TTE during	Reference	Reference	Reference	Reference
TTE before IMV	2.75 (0.002, 5.49)	0.050	2.12 (−1.16, 5.42)	0.21
TTE after IMV	1.68 (−0.73, 4.09)	0.17	2.12 (−1.04, 5.28)	0.19

^†^ Adjusted for chronic cardiac disease, APACHE II score, etiology of acute respiratory failure, BMI, COPD, and OSA.

**Table 4:** Echocardiography parameters among participants with LVEF ≥ 55%.

N = 291	Total Cohort (N = 291)	TTE-IMV (N = 161)	TTE-bIMV (N = 57)	*p*-Value ^1^ (IMV vs. bIMV)	TTE-aIMV (N = 73)	*p*-Value ^1^ (IMV vs. aIMV)
LVGLS *	(n = 282)	(n = 160)	(n = 53)		(n = 69)	
Median (Q1–Q3)	17.9 (14.1–21.8)	16.6 (13.2–19.6)	19.8 (17.1–24.2)	<0.001	19.6 (16.2–23.4)	<0.001
LVEF						
Median (Q1–Q3)	65 (60–70)	65 (60–70)	65 (60–70)	0.67	65 (60–65)	1.00

^1^ Kruskal–Wallis test with Bonferroni correction for multiple comparisons. * Absolute strain values are reported with lower values indicating worse strain.

**Table 5:** Unadjusted and adjusted analysis for LVGLS among participants with LVEF ≥ 55%.

	Unadjusted β (95% CI)	*p*-Value	Adjusted β ^†^ (95% CI)	*p*-Value
LVGLS				
TTE-IMV	Reference	Reference	Reference	Reference
TTE-bIMV	3.34 (1.68, 4.99)	<0.001	4.18 (2.22, 6.15)	<0.001
TTE-aIMV	3.05 (1.59, 4.51)	<0.001	3.45 (1.50, 5.40)	<0.001

^†^ Adjusted for chronic cardiac disease, APACHE II score, etiology of acute respiratory failure, BMI, COPD, and OSA.

## Data Availability

The data used in the analysis are available upon request from the corresponding authors.

## Supplementary Materials

The following supporting information can be downloaded at https://www.mdpi.com/article/10.3390/jcdd11110339/s1: Table S1: Echocardiography parameters among participants with LVEF < 55%.
