# Supplementary material for: Comparison of Left Ventricular Global Longitudinal Strain and Left Ventricular Ejection Fraction in Acute Respiratory Failure Patients Requiring Invasive Mechanical Ventilation
Source: J Cardiovasc Dev Dis. 2024 Oct 24;11(11):339. doi: 10.3390/jcdd11110339 (PMC11594607; doi:10.3390/jcdd11110339)
Supplement: Supplementary file 1 [file jcdd-11-00339-s001.zip › jcdd-3196481-supplementary.pdf]

Title: Comparison of Left Ventricular Global Longitudinal Strain and Left Ventricular Ejection Fraction in Acute Respiratory Failure Patients Requiring Invasive Mechanical Ventilation

Supplementary Table S1: Echocardiography parameters among participants with LVEF < 55%

N=73	Total Cohort (n=73)	TTE-IMV (n=52)	TTE-bIMV (n=9)	*p*-value (IMV vs. bIMV)	TTE-aIMV (n=12)	*p*-value (IMV vs. aIMV)
LV Global Longitudinal Strain	(n=72)	(n=52)	(n=8)		(n=12)	
Median (Q1-Q3)	12.4 (9.2-14.9)	12.2 (9.2-14.8)	12.6 (10.1-16.7)	0.23[1]	14.1 (9.2-15.8)	0.19[1]
LVEF						
Median (Q1-Q3)	45 (40-50)	45 (40-50)	45 (40-50)	0.47[1]	45 (42.5-45)	0.37[1]

[1] Kruskal–Wallis test with Bonferroni correction for multiple comparisons. *Absolute strain values are reported with lower values indicating worse strain.
